# Fifteen-day topical ketorolac tromethamine, with and without benzalkonium chloride, alters tear function, goblet cell density, and meibomian gland integrity in healthy cats

**DOI:** 10.14202/vetworld.2026.310-323

**Published:** 2026-01-25

**Authors:** Bruna Carvalho Silveira, Alexandre Pinto Ribeiro, Matheus Anthony Mendes, Maria Gabriela de Mendonça Mazetti, Douglas Lisboa Ramalho, Anderson Oliveira Souza, Nathalia de Assis Pereira, Nataliê Ecker

**Affiliations:** 1 Postgraduate Program in Veterinary Science, Faculty of Veterinary Medicine, Federal University of Mato Grosso, Fernando Correa da Costa Avenue, 2367, Boa Esperança, Cuiabá, 78.060-900, Mato Grosso, Brazil; 2 Faculty of Veterinary Medicine, Federal University of Mato Grosso, Fernando Correa da Costa Avenue, 2367, Boa Esperança, Cuiabá, 78.060-900, Mato Grosso, Brazil; 3 Postgraduate Program in Health Sciences, Faculty of Chemistry, Federal University of Mato Grosso, Fernando Correa da Costa Avenue, 2367, Boa Esperança, Cuiabá, 78.060-900, Mato Grosso, Brazil; 4 Faculty of Chemistry, Federal University of Mato Grosso, Fernando Correa da Costa Avenue, 2367, Boa Esperança, Cuiabá, 78.060-900, Mato Grosso, Brazil

**Keywords:** benzalkonium chloride, feline ophthalmology, goblet cell density, Ketorolac tromethamine, matrix metalloproteinase-9, meibomian gland loss, ocular surface disease, oxidative stress biomarkers, Schirmer tear test, tear film break-up time

## Abstract

**Background and Aim:**

Topical nonsteroidal anti-inflammatory drugs are commonly used in feline ophthalmology, especially for long-term management of uveitis after cataract surgery. However, there is very limited data on how they affect the feline ocular surface, particularly the conjunctival tissue, goblet cell density (GCD), meibomian glands (MGs), and oxidative stress. This study assessed whether 15-day, thrice-daily application of 0.45% preservative-free ketorolac tromethamine (FKT) or 0.4% benzalkonium chloride (BAC)–preserved ketorolac tromethamine (BACKT) influences ocular surface disease scores, tear film parameters, GCD, MG morphology, matrix metalloproteinase-9 (MMP-9), and oxidative stress biomarkers (OSB) in healthy cats.

**Materials and Methods:**

A prospective, randomized, double-masked, crossover design was used with 13 healthy cats. Each cat received FKT in one eye and BACKT in the other eye every 8 h for 15 days, followed by a 3-week washout period and reversal of treatment. A separate control group (CG; n=13) received topical saline. Clinical assessments included conjunctival hyperemia, blepharospasm, Schirmer tear test (STT), tear film break-up time (TFBT), lissamine green, and fluorescein staining. Meibography was used to quantify MG loss. Conjunctival biopsies obtained at baseline and day 15 were analyzed for GCD, MMP-9, superoxide dismutase, catalase, reduced glutathione, and malondialdehyde levels.

**Results:**

No groups showed corneoconjunctival staining or conjunctival hyperemia at any point. Mild blepharospasm developed in 3 out of 13 FKT-treated eyes and 9 out of 13 BACKT-treated eyes (p = 0.003). STT values significantly decreased from baseline to day 15 in both FKT and BACKT groups (p < 0.05). TFBT decreased significantly only in FKT-treated eyes (p = 0.009), although BACKT showed a similar, non-significant trend. MG loss increased significantly only in BACKT-treated eyes (p = 0.04). GCD decreased markedly in both FKT (p = 0.0003) and BACKT (p < 0.0001) groups and was lower than CG at day 15. OSB remained largely unchanged, except for higher MDA levels in BACKT-treated eyes compared with CG (p = 0.04). MMP-9 expression did not differ within or between groups.

**Conclusion:**

Both ketorolac formulations reduced STT, TFBT, and GCD, supporting the development of a qualitative dry eye state in healthy cats. BACKT resulted in greater ocular discomfort, increased MG loss, and higher lipid peroxidation, indicating BAC-related cytotoxicity. Caution is advised when prescribing prolonged topical ketorolac, and concurrent ocular lubrication is recommended.

## INTRODUCTION

Topical nonsteroidal anti-inflammatory drugs (NSAIDs) are commonly used in veterinary ophthalmology to manage anterior segment inflammation in cats and are often preferred for diabetic patients who need careful perioperative management [1–3]. Among these drugs, ketorolac tromethamine (KT) is a frequently administered non-selective ophthalmic NSAID. Research has shown that even a single drop of KT effectively reduces prostaglandin E_2_ levels after paracentesis-induced disruption of the blood–aqueous barrier in cats [3].

Benzalkonium chloride (BAC), which is found in nearly 70% of ophthalmic formulations, is well known for its cytotoxic effects on corneal and conjunctival epithelial cells and is a major factor in ocular surface disease (OSD). This condition is characterized by increased ocular surface staining, lower Schirmer tear test (STT) values, shortened TFBT, and higher conjunctival hyperemia and blepharospasm scores. Although these adverse effects are typically linked to chronic exposure, such as in glaucoma treatment, they can develop after just 7 days of BAC-containing instillations [4]. Studies involving human and canine corneoconjunctival epithelial cells have consistently shown greater cytotoxicity with BAC-preserved solutions compared to preservative-free options [5, 6]. Importantly, KT is manufactured in both BAC-preserved and preservative-free versions [7]

Local adverse reactions commonly associated with ophthalmic NSAIDs include conjunctival stinging, burning, hyperemia, and transient corneal anesthesia, while corneal melting is considered rare [8]. In healthy human volunteers, three-week instillation of KT resulted in less conjunctival lissamine green staining than bromfenac, and neither drug caused corneal epithelial injury as assessed by fluorescein staining [9]. In cats, a seven-day course of diclofenac did not affect STT, TFBT, fluorescein uptake, corneal sensitivity, pupil diameter, or intraocular pressure, although conjunctival hyperemia was significantly higher than with placebo [1].

Meibomian glands (MGs), which extend perpendicularly along the palpebral conjunctiva, play an essential role in tear film stability by producing the lipid layer that prevents evaporative loss. Infrared metrography enables non-invasive visualization of MG structure. In healthy dogs, 30 days of twice daily preservative-free KT (FKT) or BAC-preserved KT (BACKT) did not induce conjunctival hyperemia or blepharospasm but significantly shortened non-invasive TFBT and increased MG loss, with MG alterations persisting even 60 days after treatment cessation [7].

Matrix metalloproteinases (MMPs) are essential mediators of epithelial and stromal remodeling in both healthy and diseased ocular surfaces. MMP-9 is normally expressed in feline tears, corneoconjunctival epithelium, goblet cells, and MGs, and it increases significantly after experimental corneal injury [10, 11]. Evidence on how NSAID-induced modulation of MMP expression affects this process is conflicting. Diclofenac and FKT have been shown to upregulate MMP-1, -2, and -8 in intact rat corneas, suggesting potential cytotoxicity [12], whereas other studies report no significant effect of FKT on MMP-9 expression in injured rabbit corneas [13].

Oxidative stress indicators, including catalase (CAT), superoxide dismutase (SOD), glutathione (GSH), and malondialdehyde (MDA), have been reported to fluctuate in response to ocular disease or inflammation across multiple species [3, 7, 14–20]. In dogs, long-term KT instillation reduced conjunctival goblet cell density (GCD), shortened TFBT, and increased MG loss, with BAC-containing formulations additionally decreasing SOD and raising MDA levels [7].

In feline practice, topical NSAIDs are often used for long periods, especially after cataract surgery and in managing chronic uveitis [21, 22]. Despite this, only one study has looked at ocular surface changes following short-term diclofenac use in cats, and conjunctival tissue was not examined [1]. Although five-day KT administration did not change corneal sensitivity in healthy cats [2], the longer-term effects of KT, particularly on OSD scores, MG morphology, and conjunctival physiology, still remain unknown.

Despite the widespread use of topical NSAIDs in feline ophthalmology, especially in managing chronic uveitis and post-cataract surgery care, there is still a significant lack of evidence about their safety on the feline ocular surface. Earlier studies mainly focused on short-term use or examined only limited clinical parameters like tear quantity or corneal sensitivity, without exploring stronger structural and biochemical effects. Notably, the only feline study on NSAID-induced ocular surface changes involved a brief 7-day diclofenac protocol and did not include histological analysis of conjunctival tissue, GCD, MG structure, oxidative stress biomarkers (OSB), or MMPs. Additionally, although the toxic effects of BAC, a common preservative in ophthalmic solutions, have been well documented in humans, dogs, and cell culture models, these findings cannot be directly applied to cats due to species-specific differences in tear composition, conjunctival architecture, MG function, and oxidative stress responses. No published research has systematically compared the ocular effects of FKT versus BACKT in cats, nor has any study assessed whether these formulations cause subtle or early signs of OSD, such as GCD reduction, MG loss, lipid peroxidation, or changes in tear film quality, during relevant treatment durations. This creates a vital knowledge gap, especially since cats often require NSAID therapy for weeks or months in routine clinical practice.

This study aimed to thoroughly assess the effects of topical KT on the ocular surface in healthy cats by comparing FKT and BACKT formulations, administered three times daily for 15 days. Specifically, it sought to determine whether these treatments change clinical indicators of OSD, including STT, tear film break-up time (TFBT), conjunctival hyperemia, and blepharospasm, as well as tissue-based parameters such as conjunctival GCD, MG morphology assessed via infrared meibography, MMP-9 expression, and OSB (SOD, CAT, GSH, and MDA). Combining clinical, morphological, histological, and biochemical data within a randomized, double-masked crossover design, this study offers the first comprehensive overview of ketorolac-related ocular surface changes in cats. The results are intended to support evidence-based decisions, promote safe therapeutic approaches, and highlight potential risks related to short-term and long-term NSAID use in feline ophthalmology.

## MATERIALS AND METHODS

### Ethical approval

This study was conducted following the Guide for the Care and Use of Animals in Research and Teaching and received approval from the Ethics Committee of the Federal University of Mato Grosso (UFMT) 23108.032300/2022-79. All examined and biopsied cats were privately owned and belonged to veterinary staff members of the UFMT Veterinary Teaching Hospital. Written informed consent was obtained from the owners before including the cats in the study. All clinical and ophthalmological examinations were performed in accordance with good veterinary practice guidelines. Painful clinical procedures, such as conjunctival biopsies performed in both groups (described below), were carried out under topical anesthesia.

### Study period and location

This study was conducted from April 2022 to March 2025, which included data selection, clinical evaluation, and analytical procedures. The study was conducted at the Veterinary Hospital of the Federal University of Mato Grosso, located in Cuiabá, Mato Grosso, Brazil.

### Study design

Twenty-six healthy, short-haired, domestically owned cats were included in this study. The exclusion criteria were recent (<3 weeks) or current treatment with any systemic or topical ophthalmic medications, and the presence of ophthalmic or other systemic health conditions. Blood samples were taken for hematological, biochemical (including alanine aminotransferase, albumin, and blood urea nitrogen), and molecular tests. Only cats with no abnormalities detected during physical, ocular, hematological, or biochemical exams were included. The selected cats were dewormed (Endogard, Virbac, Brazil), housed, and acclimated to the examiners and procedures for 2 days in a climate-controlled environment (39 lux light, 42.5% humidity, 25°C). They were exposed to a 12-h light/dark cycle, fed dry cat pellet diet twice daily, and had free access to water.

A prospective, randomized, double-masked, crossover study was conducted with 13 cats (6 males and 7 females). An additional group of 13 cats (6 males and 7 females) received saline in both eyes every 8 h for 15 consecutive days and served as the control group (CG). Cats in the CG were not included in the crossover design because they received no other treatment and were not subjected to a second trial. Treatments were randomly assigned using an online randomization tool (http://www.randomization.com).

### Treatment groups and administration of drugs

In the crossover group (n = 13), one eye was treated with 40 μL of 0.45% FKT ophthalmic solution (pH 6.8) (Acular® CMC, Allergan, Guarulhos, Brazil), while the contralateral eye received 40 μL of 0.4% BACKT ophthalmic solution (pH 7.4) (Acular® LS, Allergan, Guarulhos, Brazil), every 8 h for 15 consecutive days. After a 3-week washout period, each cat received the alternate formulation in each eye; that is, the eye previously treated with FKT received BACKT, and vice versa, and the process was repeated. Cats in the CG (n = 13) received 40 μL of sterile saline solution (Solução fisiológica, Soluflex®, Halex Istar, Goiânia, Brazil) in both eyes every 8 hours for 15 days and did not undergo a second trial because they did not receive any other treatment.

For proper administration, the same observer pipetted ophthalmic solutions and saline and instilled them. The cats were restrained in a lying position with their heads elevated, and the eyelids were gently opened to ensure that the entire drop reached the ocular surface. Before each instillation, the cats’ eyelids were wiped to remove debris. The operator’s hands were washed with water and conventional soap between administrations to prevent cross-contamination.

### Clinical ophthalmic examinations

All ophthalmic assessments were performed to establish baseline values before treatment was administered and were repeated 15 days after starting treatment. All examinations were conducted by the same examiner, who was blinded to the treatments, at fixed times (8:00 a.m. and 5:00 p.m.) to reduce inter-observer variability. Additionally, all evaluations occurred in a controlled environment with an illumination level of 39 lux, a relative humidity of 42.5%–45%, and a temperature of 25°C (Medidor Multifunção ITPM-600, Instrutemp, Uberlândia, Brazil).

### Blepharospasm and conjunctival hyperemia

Blepharospasm was assessed as an indicator of ocular discomfort, while conjunctival hyperemia was evaluated as an indicator of ocular surface inflammation, as previously described [11]. Clinical signs were graded as absent (0), mild (1), moderate (2), or severe (3) using a previously validated scoring system [11].

### STT and TFBT

The STT Strips, Ophthalmos, São Paulo, Brazil) was used to measure the tear film’s aqueous layer [23]. The TFBT (Fluorescein Strips, Ophthalmos, São Paulo, Brazil) was employed to provide a subjective assessment of the tear film’s stability and quality [24].

### Corneal and conjunctival staining

Fluorescein and lissamine green staining were used to evaluate the integrity and health of the corneoconjunctival surface [25]. The staining intensity was rated as absent (0), mild (1), moderate (2), or severe (3), based on previously validated criteria [25].

### Meibography

**Figure 1:**
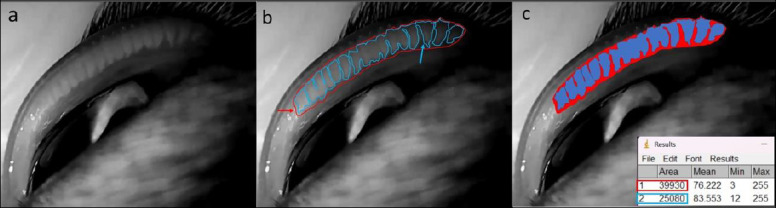
Workflow for quantification of meibomian gland (MG) loss using infrared meibography and ImageJ analysis. (a) Representative meibography image of the feline palpebral conjunctiva after adjustment of brightness and contrast to enhance MG visualization. (b) Delineation of the total MG reference area (Area 1; red outline and arrow), defined as 100% of the MG region, followed by manual tracing of the MG-filled area (Area 2; blue outline and arrow) using the freehand selection tool. (c) ImageJ output illustrating the calculated areas and the derived percentage of MG loss, shown in a representative control eye at baseline (T0), where MG loss corresponded to 37.19%. MG loss was calculated as: [(Total MG area − MG-filled area) × 100] / Total MG area.

After selection, the cats were anesthetized, the lights were turned off, and the upper eyelids were everted. Non-contact infrared meibography was then performed, and the MG area was recorded using a security camera (Cs-C1C, EZVIZ®, Hangzhou, China). The best image capturing the entire palpebral conjunctival area was chosen, and video screenshots were taken (Figure 1). The images were analyzed with ImageJ software (http://www. rsbweb. nih.gov/ij/). All measurements were performed twice after clinical ophthalmic evaluations by the same examiner in the same room and at the same time points.

Images were anonymized for evaluation using a randomization website (http://www.randomization.com). Two independent observers, blinded to the patient groups, analyzed the images using ImageJ. A second review was performed by the same observers 2 weeks after the initial assessment.

During evaluation, brightness and contrast were adjusted independently to improve visualization of the MG boundary. Using the freehand tool, the entire MG region (area 1) was outlined and set as 100% of the reference area. The MG-filled region (area 2) was then outlined, and MG loss (%) was calculated as:

MG loss (%) = (Total area − MG area) × 100 / Total area.

### Conjunctival biopsy and GCD determination

Two conjunctival biopsies (3.0–4.0 mm) were collected from the same eye using previously described methods [7, 26]. Half of each biopsy was used to determine GCD, and the remaining portion was stored at −80°C in 1% protease inhibitor cocktail for oxidative stress and MMP-9 analyses.

Baseline samples were collected from the dorsomedial fornix one week before treatment, and day-15 samples were obtained from the ventromedial fornix one hour after the final treatment. Tissues were processed routinely and stained with Periodic Acid–Schiff staining and hematoxylin-eosin. At ×40 magnification, the nuclei of 50 adjacent basal epithelial cells were identified, and goblet cells within the same fields were counted [7]. All analyses were conducted by a single blinded examiner.

### Quantification of OSB

Stored conjunctival samples were thawed and homogenized in buffer solution with protease and phosphatase inhibitors. Protein concentration was determined using Bradford’s test.

- SOD activity was assessed using the established method [27] with modifications. SOD activity was assessed using a modified epinephrine auto-oxidation inhibition method. Enzymatic activity was quantified based on the capacity of the samples to inhibit the rate of epinephrine auto-oxidation relative to a blank (sample-free) control, under standardized pH, temperature, reaction time, and final reaction volume conditions.
- CAT activity was measured using Tris–HCl buffer and H_2_O_2_ extinction coefficients [28].
- GSH levels were measured using the o-Phthalaldehyde reaction [29].
- MDA levels were determined using the thiobarbituric acid reaction and spectrophotometry at 535 nm [30].

All assays were performed in duplicate and analyzed blindly.

### Quantification of MMP-9 expression

Equal amounts of protein were used for all samples and analyzed with a commercial enzyme-linked immunosorbent assay (ELISA) kit (Matrix CAt®, MyBioSource®, Canada). Standards, blanks, and positive controls were processed according to the manufacturer’s protocol. Absorbance was measured at 450 nm, and MMP-9 concentrations were calculated using four-parameter logistic modeling (MyAssays). Detection limits ranged from 10 to 320 ng/mL. Coefficients of variation were less than 8% (intra-assay) and less than 10% (inter-assay). All measurements were conducted blindly.

### Statistical analysis

Sample size was calculated using Sealed Envelope with α = 5% and 80% power, based on previously reported outcome variability in cats and dogs [1, 7, 26]. The Shapiro–Wilk test was used to assess normality. Student’s t-tests were used to compare age and weight between groups. One-way analysis of variance with Bonferroni correction was used to compare treatment groups at each time point. Paired t-tests assessed within-group changes from baseline to day 15. A significance level of p < 0.05 was adopted. Statistical analyses were conducted using R (R Core Team, Vienna, Austria, 2024).

## RESULTS

### Animals

The mean ± SD age of cats in the KT group (1.69 ± 0.85) and the CG group (2.00 ± 0.81) did not differ significantly between groups (p = 0.35). The mean ± SD weight of cats in the KT group (3.50 ± 0.57) and the CG group (3.15 ± 0.82) did not differ significantly (p = 0.21).

### Clinical signs and ophthalmic findings

Comparisons within each time point showed no significant differences in STT and TFBT values at baseline or 15 days after treatments (p > 0.05) (Table 1) [23, 31]. STT values significantly decreased from baseline to day 15 in both FKT and BACKT (p < 0.05), while no significant changes occurred in CG (p = 0.66). A significant decrease in TFBT was observed from baseline to day 15 only in the eyes that received FKT (p = 0.009) (Table 1).

**Table 1:** Tear film parameters and ocular discomfort scores in healthy cats treated with saline, preservative-free ketorolac tromethamine, or benzalkonium chloride–preserved ketorolac tromethamine.

Parameters and time points	CG (n = 13)	FKT (n = 13)	BACKT (n=13)	p-value among treatments
STT (mm/min)				
T0	17.15 ± 4.48 (14.44–19.87)	19.38 ± 4.03 (16.95–21.82)	19.00 ± 5.27 (15.81–22.19)	0.43
T1	16.85 ± 4.05 (14.39–19.30)	16.00 ± 6.81 (11.88 to 20.12)	16.08 ± 4.80 (13.17–18.98)	
p-value between time points	0.66	0.01	0.04	0.90
TFBT (seconds)			1	
T0	16.00 ± 2.23 (14.65–17.35)	18.92 ± 5.07 (15.86–21.99)	9.23 ± 6.72 (15.17–23.29)	0.20
T1	15.92 ± 2.36 (14.50–17.35)	14.69 ± 4.11 (12.21–17.18)	14.23 ± 3.78 (11.94–16.52)	
p-value between time points	0.87	0.009	0.08	0.45
Blepharospasm				
T0	0.00	0.00	0.00	–
T1	0.00	0.23 ± 0.43^a^(–0.03 to 0.49)	0.69 ± 0.48^b,c^(0.40 to 0.98)	< 0.0001^a,b^,
p-value between time points	–	0.25	0.003	0.003^c,c^

Values are presented as mean ± standard deviation with 95% confidence intervals. T0 = baseline, T1 = day 15. Different superscript letters (a–c) within the same row indicate statistically significant differences among treatment groups at the same time point (p < 0.05). Within-group p-values denote comparisons between T0 and T1. Reference ranges: Schirmer tear test (STT) 16.50–18.51 mm/min [23]; tear film break-up time (TFBT) 9.1–17.7 s [31]. Statistical significance was set at p < 0.05.

Fluorescein and lissamine green staining were absent at both time points in all groups. Conjunctival hyperemia was not observed over time in either the control or treatment groups. Comparison with the baseline showed that blepharospasm increased from absent to mild in both FKT and BACKT groups on day 15, but significant differences were found only in the BACKT group (p < 0.0001) (Table 1). Additionally, when comparing groups on day 15, BACKT showed significantly higher blepharospasm levels than CG (p < 0.0001) and FKT (p = 0.003) (Table 1).

### OSB

**Figure 2:**
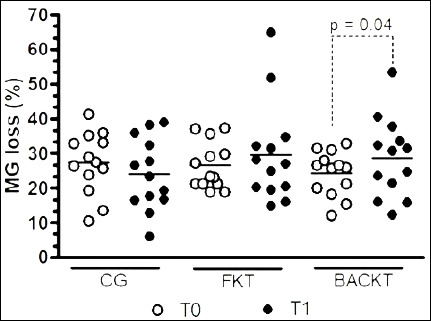
Changes in meibomian gland (MG) loss over time in eyes treated with saline (control group, CG), preservative-free ketorolac tromethamine (FKT), and benzalkonium chloride–preserved ketorolac tromethamine (BACKT). Data represent MG loss (%) measured by infrared meibography at baseline (T0) and after 15 days of treatment (T1). Lines indicate group means, and symbols represent individual eyes. Quantification was performed using ImageJ software.

Comparisons between timepoints (T0 vs. T1) within each group showed no significant differences in the levels of SOD, CAT, GSH, and MDA (p > 0.05) (Table 2; Figure 2). No significant differences in SOD, CAT, and GSH activity were observed among treatment groups at either time point (p > 0.05) (Table 2; Figure 2). At T1, MDA levels were significantly higher in the BACKT group compared to the CG (p = 0.04) (Table 2; Figure 2).

**Table 2:** Conjunctival oxidative stress biomarkers and matrix metalloproteinase-9 levels in healthy cats following topical ketorolac treatment.

Parameters and time points	CG (n = 13)	FKT (n = 13)	BACKT (n=13)	p-value among treatments
Catalase (U)				
T0	3.23 ± 2.12 (1.88 to 4.58)	2.22 ± 1.05 (1.26 to 3.18)	2.46 ± 1.26 (1.66 to 3.27)	0.31
T1	2.68 ± 2.56 (1.05 to 4.30)	1.58 ± 0.99 (0.94 to 2.21)	1.64 ± 1.35 (0.78 to 2.50)	
p-value between time points	0.64	0.27	0.20	0.24
GSH (mmol/L)				
T0	15.47 ± 0.36 (15.24–15.70)	13.47 ± 2.16 (12.09–14.85)	14.60 ± 2.91 (12.75–16.45)	0.08
T1	15.84 ± 1.31 (15.00 to 16.67)	15.02 ± 3.40 (12.86–17.18)	15.09 ± 3.90 (12.60–17.57)	
p-value between time points	0.26	0.06	0.64	0.77
MDA (µmol/g)				
T0	7.30 ± 11.45 (0.02–14.58)	6.76 ± 4.05 (4.19–9.34)	8.97 ± 4.76 (5.94 to 12.00)	0.07
T1	5.58 ± 6.95^a^(1.16 to 10.00)	6.60 ± 3.98 (4.07–9.14)	10.72 ± 7.79^b^(5.77–15.67)	
p-value between time points MMP-9 (ng/mL)	0.26	0.85	0.23	0.04
T0	115.9 ± 12.3 (108.5 to 123.4)	109.2 ± 19.2 (97.63–120.8)	114.12 ± 12.86 (106.5 to 122.1)	0.50
T1	108.0 ± 12.45 (100.5 to 115.5)	105.7 ± 12.08 (98.42–113.0)	104.9 ± 9.06 (99.47–110.4)	
p-value between time points	0.06	0.61	0.07	0.77

Values are expressed as mean ± standard deviation with 95% confidence intervals. T0 = baseline, T1 = day 15. SOD = superoxide dismutase, CAT = catalase, GSH = reduced glutathione, MDA = malondialdehyde, MMP-9 = matrix metalloproteinase-9, CG = control group, FKT = preservative-free ketorolac tromethamine, BACKT = benzalkonium chloride–preserved ketorolac tromethamine. Different superscript letters (a,b) within the same row indicate statistically significant differences among treatment groups at the same time point (p < 0.05). Within-group p-values refer to comparisons between T0 and T1. Statistical significance was set at p < 0.05.

### MMP-9

No significant differences in MMP-9 were observed within or between the treatment groups at either T0 or T1 (p > 0.05) (Table 2; Figure 2).

### Meibography

Interobserver agreement in 10 MG images showed significant correlations between the first and second annotations for both observers (p = 0.001) (Table 3). Bland-Altman analysis indicated that observer 1 tended to underestimate MG loss, while observer 2 tended to overestimate it (Table 3). Annotations between observers 1 and 2 (intra-observer agreement) also demonstrated significant correlations (p < 0.0001) (Table 4). Bland-Altman analysis revealed that observer 2 tended to overestimate MG loss compared to observer 1 (Table 4).

**Table 3:** Inter-observer repeatability of meibomian gland (MG) loss measurements obtained from infrared meibography.

Statistical parameters	Observer 1	Observer 2
R Person (95% confidence interval)	0.80 (0.4256 to 0.9427)	0.81 (0.4631–0.9476)
Bias ± Standard deviation	–1.46 ± 4.91	4.08 ± 7.26

Agreement between the first and second annotations performed by each observer was assessed in a subset of control and treated eyes using Pearson's correlation coefficient (95% confidence interval) to evaluate linear association and Bland–Altman analysis to determine measurement bias and limits of agreement. These analyses describe the consistency and reliability of repeated MG loss quantification using ImageJ. Agreement bias and variability between measurements are expressed as mean difference ± standard deviation. MG = meibomian gland. Statistical significance was set at p < 0.05.

**Table 4:** Intra-observer agreement between two independent observers for the quantification of MG loss using infrared meibography.

Parameter	R Pearson (95% Confidence interval)	Bias ± Standard deviation
Meibomian gland loss	0.90 (0.6887–0.9734)	3.99 ± 4.44

Intra-observer agreement refers to the consistency between measurements obtained by Observer 1 and Observer 2 for the same images. Pearson's correlation coefficient (r) with 95% confidence intervals was used to evaluate the strength of association between observers. Bland–Altman bias is expressed as mean difference ± standard deviation, indicating systematic differences and measurement variability. MG = meibomian gland. Statistical significance was set at p < 0.05.

**Figure-3:**
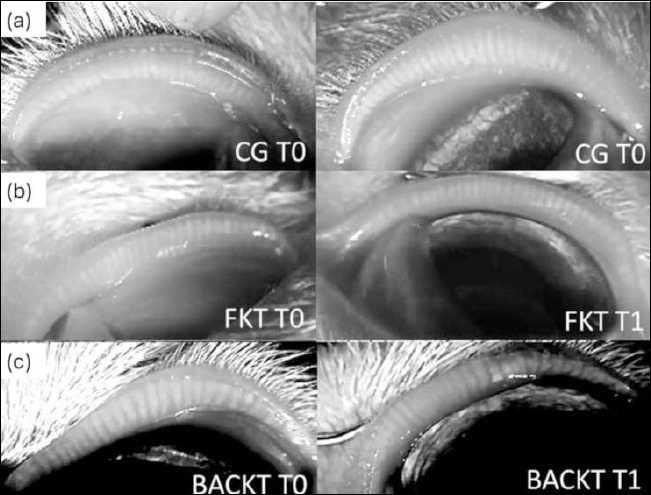
Representative infrared meibography images of the palpebral conjunctiva showing meibomian gland (MG) morphology at baseline (T0) and after 15 days of treatment (T1). Images correspond to eyes treated with (a) saline (control group, CG), (b) preservative-free ketorolac tromethamine (FKT), and (c) benzalkonium chloride–preserved ketorolac tromethamine (BACKT). Images illustrate qualitative changes in MG structure across treatment groups and time points. All images were captured and analyzed using ImageJ software to evaluate gland structure and loss.

The lowest and highest percentages of MG loss observed at T0 were 10.40% and 41.21%, respectively. At T1, the lowest and highest percentages were 6.04% and 64.86%, respectively. The average MG loss did not differ among the groups at either T0 or T1 (p > 0.05) (Table 5; Figures 2 and 3). Regarding comparisons between timepoints, a significantly higher MG loss was observed only in the eyes that underwent BACKT (p = 0.04).

**Table 5:** Quantitative assessment of MG loss in healthy cats following topical ketorolac administration.

Time points	CG (n = 13)	FKT (n = 13)	BACKT (n = 13)	p-value
T0	27.20 ± 8.90 (21.82–32.59)	26.50 ± 6.80 (22.39–30.61)	24.25 ± 6.41 (20.37–28.12)	0.57
T1	24.04 ±10.33 (17.80–30.29)	29.75 ± 14.35 (21.08 to 38.42)	28.81 ± 11.44 (21.90–35.72)	0.51
p-value	0.40	0.48	0.04	

Values are expressed as mean ± standard deviation with 95% confidence intervals. T0 = baseline, T1 = day 15. MG = meibomian gland, CG = control group, FKT = preservative-free ketorolac tromethamine, BACKT = benzalkonium chloride–preserved ketorolac tromethamine. MG loss is presented as the percentage of glandular area loss relative to the total meibomian gland reference area, quantified using infrared meibography and ImageJ analysis. Within-group p-values indicate comparisons between T0 and T1. Statistical significance was set as p < 0.05.

### GCD

**Figure 4:**
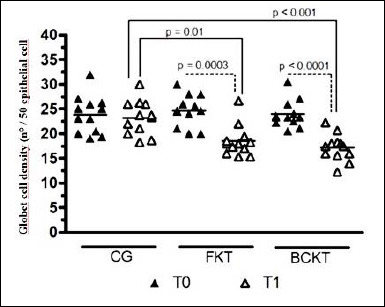
Conjunctival goblet cell density assessed at baseline (T0) and after 15 days of treatment (T1) in eyes treated with saline (control group, CG), preservative-free ketorolac tromethamine (FKT), and benzalkonium chloride–preserved ketorolac tromethamine (BACKT). GCD is expressed as the number of goblet cells per 50 conjunctival epithelial cells. Lines represent group means, and symbols indicate individual values.

**Figure 5:**
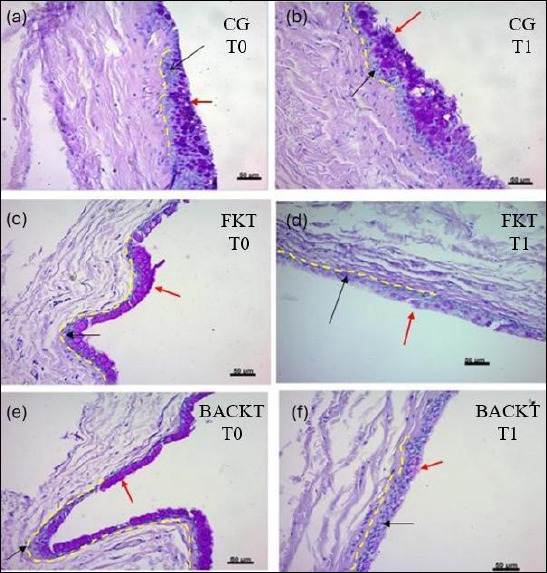
Representative histological sections of feline conjunctiva illustrating goblet cell density (GCD) at baseline and after 15 days of treatment. (a and b) Saline-treated control eyes at T0 and T1, (c and d) preservative-free ketorolac tromethamine (FKT)–treated eyes at T0 and T1, and (e and f) benzalkonium chloride–preserved ketorolac tromethamine (BACKT)–treated eyes at T0 and T1. Goblet cells (red arrows) were counted among 50 adjacent conjunctival epithelial cells (black arrows). The dashed line outlines the counting area. Sections were stained with Periodic acid–Schiff/hematoxylin and eosin; original magnification ×40. The second biopsy was obtained from a different conjunctival region to avoid scar tissue from the initial samplin

Comparisons within each treatment group over time showed that GCD significantly decreased from T0 to T1 in both the FKT (p = 0.0003) and BACKT (p < 0.0001) groups (Table 6, Figures 4 and 5).

**Table 6:** Conjunctival goblet cell density in healthy cats following topical ketorolac treatment.

Time points	CG (n = 12)	FKT (n = 11)	BACKT (n = 11)	p-value
T0	23.84 ± 3.83 (21.41–26.27)	24.70 ± 3.17 (22.68–26.72)	23.94 ± 2.75 (22.19–25.69)	0.78
T1	23.24 ± 3.48 (21.03 to 25.46)	18.49 ± 3.18^a^(16.47 to 20.51)	17.18 ± 2.70^b^(15.46–18.90)	
p-value	0.06	0.0003	< 0.0001	0.01^a^, 0.001^b^

Values are expressed as mean ± standard deviation with 95% confidence intervals. T0 = baseline, T1 = day 15. CG = control group, FKT = preservative-free ketorolac tromethamine, BACKT = benzalkonium chloride–preserved ketorolac tromethamine. GCD = conjunctival goblet cell density, defined as the number of goblet cells per 50 directly adjacent conjunctival epithelial cells. Different superscript letters (a, b) within the same row indicate statistically significant differences compared with the control group at the same time point. Within-group p-values denote comparisons between T0 and T1. Statistical significance was set at p < 0.05.

Comparisons among treatment groups assessed at T0 showed no statistically significant differences (p = 0.78) (Table 6, Figures 4 and 5). At T1, GCD values between FKT and BACKT did not differ (p = 0.28). However, at the same time point, significantly lower values were observed when FKT (p = 0.01) and BACK (p < 0.0001) were compared with CG (Table 6, Figures 4 and 5).

## DISCUSSION

### Clinical signs and ophthalmic findings

This was the first study to assess the effects of ophthalmic instillation of an NSAID solution, with or without BAC, on ophthalmic clinical signs, meibography, conjunctival GCD, OSB, and MMP-9 quantitation in cats. In clinical practice, basic tests used to evaluate ocular surface health include the STT (a quantitative measure of tear aqueous volume), TFBT (a subjective evaluation of tear quality), and scoring systems for blepharospasm (a subjective assessment of blinking frequency in response to corneoconjunctival nerve stimulation), conjunctival hyperemia (a subjective grading of conjunctival redness related to inflammation), lissamine green staining (used to identify devitalized corneal and conjunctival epithelial cells), and fluorescein staining (used to detect corneal epithelial damage) [25].

In this study, 15-day instillation of both KT formulations, three times daily, did not change the scores of corneoconjunctival staining. Negative fluorescein staining has also been observed in cats following experimental ophthalmic instillation of diclofenac four times a day for 7 days [1]. However, the same study found that the likelihood of a cat showing at least one specific sign of ocular irritation, such as conjunctival hyperemia and blepharospasm, was 4.34% with diclofenac, compared to 1.08% with the control treatment [1]. In this study, although neither KT formulation caused conjunctival hyperemia, cats that received the BACKT solution had significantly higher short-duration blepharospasm scores, indicating a possible immediate irritant effect of this preservative in this species.

In this study, the STT and TFBT values measured across all groups and time points were within the reference range for cats of the same age and sex [23, 31]. Although no differences in either tear parameter were observed among treatment groups at any time point, a significant decrease in STT values was noted in both FKT- and BACKT-treated eyes after 15 days. Similarly, TFBT declined after 15 days, but a statistical difference was observed only in FKT-treated eyes.

Our findings differ from those of a previous study in cats, in which TFBT and STT values remained unchanged after ophthalmic instillation of diclofenac 4 times daily for 7 days [1]. Such differences may be due to the smaller sample size (n = 4) and shorter treatment duration in that study [1] compared to the 15-day period used in this investigation. Conversely, another study found that 60 days of treatment with either diclofenac or nepafenac did not alter STT or TFBT values in patients undergoing phacoemulsification [32].

The authors recognize that a 15-day evaluation period is a limitation of this study, as this duration may be too short to observe changes in this and other parameters. Consequently, further research should be carried out over longer periods, with more patients, and in a clinical setting. Our findings indicate that both KT ophthalmic solutions, with or without BAC, may decrease STT readings in cats. This is important because STT has been reported as a reliable diagnostic test when performed either in a familiar environment [23] or in calm or noisy hospital settings [24].

On day 15, a statistically significant reduction in TFBT values was confirmed only in FKT-treated eyes (4.23 s), while BACKT-treated eyes also showed a comparable decrease (–5.00 s). Both reductions exceeded the minimal change seen in saline-treated eyes (0.08 s). The lack of statistical significance in the BACKT group may be due to higher variability, as indicated by larger standard deviations and wider confidence intervals. Nonetheless, this finding could still be clinically relevant, suggesting that both solutions might impact this tear parameter.

Comparable results were observed in a study using an equivalent methodology in dogs, in which NI-TFBT was also significantly reduced after 15 days only in individuals treated with the FKT solution [7]. However, the same study [7] showed that both FK- and BACKT-treated dogs exhibited shortened NI-TFBT by day 30, suggesting that longer treatment durations may reveal similar changes.

### Meibography

To date, infrared meibography for the assessment of MGs has not been reported in cats. In dogs, reference values for this parameter have been published using an automated ocular surface analyzer [33, 34]. The use of an infrared security camera enabled assessment of upper eyelid MGs in cats, thereby allowing evaluation of these anatomical structures in resource-limited settings.

Although it requires substantial user input and manual MG tracing, ImageJ is freely available and enables assessment of MG loss [35]. Measurements of the same MGs and MG area, conducted by the same annotator at different times, showed a strong correlation coefficient. The intra-observer correlation coefficient also suggested moderate repeatability between the two annotators. To the authors’ knowledge, potential correlations and intra- and inter-observer agreement for MG loss using an automated ocular surface analyzer have not been previously reported in veterinary ophthalmology.

Meibography can be interpreted either by grade or by the percentage of MG loss as follows: grade 0 (area of loss 0%); grade 1 (area of loss ≤ 25%); grade 2 (area of loss 26%–50%); grade 3 (area of loss 51%–75%); and grade 4 (area of loss > 75%) [7, 33, 34]. Our findings showed that the baseline MG loss in the upper eyelids of cats, assessed in 39 healthy eyes before treatments (24.16%), is similar to that reported in the upper eyelids of 64 clinically healthy canine eyes (25.3%) [34]. In the present study, the average percentage of MG loss observed in cats is equivalent to grades 1 and 2, which is considered normal for healthy dogs [7, 33, 34].

In a previous study involving dogs, instillation of the same agents for 15 days resulted in significantly greater MG loss only in eyes that received FKT [7]. Although increased losses were seen in all eyes treated with KT on day 15, only the BACKT-treated eyes showed significant changes. However, the same study [7] revealed that both FK- and BACKT-treated dogs experienced significant MG loss by day 30, suggesting that longer treatment periods may yield similar effects.

To the best of our knowledge, the only study comparing the effects of FKT and BACKT ophthalmic solutions on MGs reported that after 60 days of treatment with both agents, meibography readings tended to return to baseline values [7]. This suggests that despite the harmful effect of BACKT on these structures, MG restoration may occur once instillations are discontinued. However, further studies are needed to confirm this finding.

### GCD

This study showed that GCD significantly decreased after 15 days of continuous KT application, with or without BAC. Two earlier studies in cats found that higher GCDs are similarly distributed in the dorsomedial and ventromedial conjunctival fornices [26, 36]. This indicates that the differences observed in our results are unlikely to be due to regional anatomical variation in GCD distribution and are more likely to be caused by a harmful effect of KT application.

A previous study on dogs treated with either FKT or BACKT for 30 consecutive days reported similar results [7]. Our findings also align with a study in humans, which demonstrated that even a preservative-free diclofenac ophthalmic solution used for 30 days after phacoemulsification significantly reduced conjunctival GCD [37].

Another study conducted in rabbits [38] further supports the hypothesis that the decrease in GCD observed in the present study may result from a direct harmful effect of KT [38]. In that study, rabbits treated for 30 consecutive days with various ophthalmic solutions (vasoconstrictors, hypotensive agents, antihistamines, and antibiotic/corticosteroid combinations), containing or not BAC (n = 114 eyes per group), maintained similar conjunctival GCD in both groups [38].

### OSB

Enzymatic antioxidants include SOD, CAT, and GPx, while the non-enzymatic antioxidant group includes GSH and vitamins A, C, and E. GSH works with SOD, CAT, and GPx to protect cells from reactive oxygen species (ROS) and maintain redox balance.

SOD acts as the first line of defense by converting highly reactive superoxide radicals into hydrogen peroxide, a less harmful intermediate [20, 39]. Hydrogen peroxide is then quickly neutralized by either CAT, which breaks it down into water and oxygen, or GPx, which reduces it using GSH as a cofactor. During this process, GSH is oxidized to glutathione disulfide [39]. Glutathione reductase regenerates reduced GSH from GSSG using Nicotinamide adenine dinucleotide phosphate to maintain antioxidant defenses.

The primary target of ROS is polyunsaturated fatty acids in membrane lipids, leading to lipid peroxidation and damage to cell structure and function [20]. MDA results from the peroxidation of polyunsaturated fatty acids; therefore, MDA elevation is a reliable indicator of lipid peroxidation [20].

Abnormal oxidative stress behavior, characterized by decreased SOD, CAT, and GSH levels and increased MDA levels, has been observed in the conjunctival and corneal tissues of mice and rabbits injured by ultraviolet light and fungal keratitis (Aspergillus flavus), in the tears of dogs and cats with different types of corneal ulcers, in the conjunctiva of dogs, and in the aqueous humor of cats with uveitis following experimental treatment with KT [3, 7, 14, 16, 17, 19].

In these studies, subjects treated with presumed oxidant medication or agent showed decreased levels of SOD, CAT, and GSH, and increased levels of MDA [3, 7, 14, 16, 17, 19].

No significant changes were observed in the enzymatic (SOD and CAT) or non-enzymatic (GSH) antioxidant systems in the conjunctival tissue of cats across different treatments and time points. However, significantly higher MDA levels were detected 15 days after treatment in eyes treated with BACKT compared with the other two groups. This finding supports, as with other parameters evaluated here, that BAC may be harmful to conjunctival tissues.

BACKT instillation significantly decreased conjunctival SOD levels and increased MDA levels in dogs [7]. The differences between our results and those reported in dogs, especially the lack of SOD and CAT reduction in the current study, may be due to the shorter treatment duration used here (15 days vs. 30 days) [7].

The assessment of SOD, CAT, and MDA in dogs with myxomatous mitral valve degeneration showed a significant increase in MDA alone, with no corresponding decrease in antioxidant enzymes such as SOD and CAT [40]. A review of the literature on the role of ROS and antioxidants in liver diseases also revealed that elevated ROS production can increase MDA levels without altering the activity of these antioxidant enzymes [41]. These findings partially explain our results.

### MMP-9

Our motivation for measuring the conjunctival levels of MMP-9 was based on observations from a previous study that indicated that instillation of an ophthalmic solution of diclofenac and FKT can increase the expression of MMP-1, -2, and -8 in intact rat corneas, suggesting that MMPs may act as markers of corneal cytotoxicity after ophthalmic NSAID administration.

However, our results showed that enzyme levels decreased after 15 days in all treatment groups, although this difference was not statistically significant.

Immunohistochemical analysis of the lacrimal gland, conjunctival-associated lymphoid tissue, and conjunctival epithelium in cats identified these tissues as the main sources of tear-derived MMP-9, with increased levels of this enzyme following corneal injury [10]. Once healing was complete and the corneal epithelium had restratified (from day 7 onward), the MMP-9 staining pattern resembled baseline levels [10].

This may, in part, explain our findings, which suggest that 15 days after conjunctivectomy, the levels of this enzyme remain unchanged regardless of KT administration.

However, it is important to note that the antibody used in the previous study [10] and the ELISA kit used in our experiment detect both latent and active forms of MMP-9. Further studies to distinguish between latent and active MMP-9 using zymography are recommended.

Additionally, our results indicate that MMP-1 and MMP-8, rather than MMP-9, may be more effective biomarkers of NSAID-related toxicity, as previously shown [12].

### Limitations

Considering that the evaluation period in this study was only 15 days and that topical NSAID instillations generally need longer durations after cataract surgery [21, 22], this could be a limitation of the current study. Regarding meibography, although Bland-Altman analysis and Pearson’s correlation were included to evaluate intra- and interobserver repeatability, the results of this study should be considered preliminary because our assessments relied on parameters typically measured by an ocular surface analyzer. Although this study justifies the number of cats included, ensuring at least 80% power to detect differences in all parameters except MMP-9, the results should be viewed with caution. Until larger studies in clinical settings are conducted, these findings should remain preliminary.

## CONCLUSION

This study offers the first comprehensive evaluation of how KT ophthalmic solutions, with and without BAC, affect the ocular surface in healthy cats. It uses a multimodal approach that includes clinical scoring, tear film assessment, meibography, conjunctival GCD, OSB, and MMP-9 measurement. Fifteen days of administering both formulations three times daily led to significant decreases in STT values. A noticeable reduction in TFBT was mainly seen in eyes treated with preservative-free ketorolac. While neither formulation caused corneal epithelial staining or conjunctival hyperemia, BAC-preserved ketorolac produced significantly greater blepharospasm, suggesting an immediate irritant effect.

Meibography showed that both solutions tended to increase MG loss, with a significant increase observed only in BAC-preserved ketorolac–treated eyes. GCD decreased significantly in both ketorolac treatments, suggesting that prolonged NSAID exposure, regardless of preservative, may negatively affect mucin-producing cells. While antioxidant defenses (SOD, CAT, GSH) remained stable, BAC-preserved ketorolac led to a significant increase in MDA levels, indicating increased lipid peroxidation and oxidative stress associated with BAC exposure. MMP-9 levels stayed consistent across all groups, suggesting that this enzyme might not be the most sensitive indicator of NSAID-related conjunctival toxicity during short treatment periods.

Practical implications include the need for clinicians to exercise caution when prescribing extended ketorolac therapy in cats, especially formulations containing BAC, due to possible effects on tear stability, mucin production, oxidative balance, and MG integrity. The findings are especially relevant for feline patients requiring long-term NSAID treatment after cataract surgery or for the management of uveitis.

Strengths of this study include its randomized, masked crossover design, multimodal assessment of ocular surface integrity, and the simultaneous evaluation of structural, functional, and biochemical outcomes.

Limitations include the brief 15-day assessment period, small sample size, and absence of long-term follow-up to assess the reversibility of MG and GCD changes. Additionally, MMP-9 analysis did not distinguish between latent and active forms.

Future research should explore longer treatment periods, study recovery after stopping therapy, differentiate between active and latent MMP-9, and include clinical feline patients with naturally occurring ocular disease to better reflect real-world conditions.

In conclusion, both preservative-free and BAC-preserved ketorolac ophthalmic solutions caused noticeable changes in feline ocular surface health, with BAC formulations causing more significant irritative and oxidative effects. These results emphasize the importance of careful use of topical NSAIDs in cats and the need for preservative-free options whenever long-term treatment is expected.

## DATA AVAILABILITY

All the generated data are included in the manuscript.

## AUTHORS’ CONTRIBUTIONS

BCS: Data curation, investigation, project administration, tutor communication, and manuscript drafting. APR: Designed the study, reviewed the manuscript, and secured funding. MAM: Assisted with sample management and collection. MGMM: Assisted with sample management and collection. DLR and ASO: Conducted laboratory analysis, including measurement of enzyme activity to assess oxidative stress. NAP: Carried out laboratory analysis, including quantification of MMP-9 using the ELISA kit. NE: Prepared histological slides and counted goblet cells. All authors have read and approved the final version of the manuscript.

## COMPETING INTERESTS

The authors declare that they have no competing interests.

## PUBLISHER’S NOTE

Veterinary World remains neutral with regard to jurisdictional claims in the published institutional affiliations.

## References

[1] Hsu KK, Pinard CL, Johnson RJ, Allen DG, KuKanich BK, Nykamp SG (2015). Systemic absorption and adverse ocular and systemic effects after topical ophthalmic administration of 0.1% diclofenac to healthy cats. Am J Vet Res.

[2] Roberts JK, Meekins JM, Roush JK, Rankin AJ (2021). Effects of topical instillation of 0.1% diclofenac sodium, 0.5% ketorolac tromethamine, and 0.03% flurbiprofen sodium on corneal sensitivity in ophthalmologically normal cats. Am J Vet Res.

[3] Miranda HR, Ribeiro AP, Pizzinatto FD, Rodrigues BE, Assis Pereira N, Oliveira Alves TH (2025). Effects of loteprednol etabonate and ketorolac tromethamine on intraocular inflammation and oxidative stress after paracentesis-induced blood–aqueous barrier breakdown in cats. Vet Ophthalmol.

[4] Goldstein MH, Silva FQ, Blender N, Tran T, Vantipalli S (2022). Ocular benzalkonium chloride exposure:problems and solutions. Eye (Lond).

[5] Hendrix DV, Ward DA, Barnhill MA (2002). Effects of antiinflammatory drugs and preservatives on morphologic characteristics and migration of canine corneal epithelial cells in tissue culture. Vet Ophthalmol.

[6] Ayaki M, Iwasawa A, Soda M, Yaguchi S, Koide R (2010). Cytotoxicity of five fluoroquinolone and two nonsteroidal anti-inflammatory benzalkonium chloride-free ophthalmic solutions in four corneoconjunctival cell lines. Clin Ophthalmol.

[7] Sonego DA, Ribeiro AP, Dower NMB, Rodrigues BE, França Lemes SA, Oliveira Souza A (2024). Effects of topical ketorolac tromethamine on tear parameters, meibography, goblet cell density, and conjunctival oxidative stress in healthy dogs. Vet Ophthalmol.

[8] Rigas B, Huang W, Honkanen R (2020). NSAID-induced corneal melt:clinical importance, pathogenesis, and risk mitigation. Surv Ophthalmol.

[9] Chu YR, Sell M (2006). Clinical evaluation of ocular surface toxicity of ketorolac tromethamine 0.4% (Acular LS®) vs bromfenac 0.09% (Xibrom®) ophthalmic solution. Invest Ophthalmol Vis Sci.

[10] Petznick A, Madigan MC, Garrett Q, Sweeney DF, Evans MD (2013). Contributions of ocular surface components to matrix-metalloproteinases-2 and -9 in feline tears following corneal epithelial wounding. PLoS One.

[11] Pizzinatto FD, Ribeiro AP, Silveira BC, Lobo PM, Miranda HR, Nathalia N (2023). Induced corneal ulcers in cats:effects of 2% dorzolamide on epithelization time and on the expression of matrix metalloproteinase-9. Acta Sci Vet.

[12] Reviglio V. E, Rana T. S, Li Q. J, Ashraf M. F, Daly M. K, O'Brien T. P ((2003)). Effects of topical nonsteroidal antiinflammatory drugs on the expression of matrix metalloproteinases in the cornea.. J Cataract Refract. Surg.

[13] Lima TB, Ribeiro AP, Conceição LF, Bandarra M, Manrique WG, Laus JL (2015). Ketorolac eye drops reduce inflammation and delay re-epithelization in response to corneal alkali burn in rabbits, without affecting iNOS or MMP-9. Arq Bras Oftalmol.

[14] Demir U, Demir T, Ilhan N (2005). The protective effect of alpha-lipoic acid against oxidative damage in rabbit conjunctiva and cornea exposed to ultraviolet radiation. Ophthalmologica.

[15] Chen BY, Lin DP, Chang LS, Huang TP, Liu HJ, Luk CP (2013). Dietary α-lipoic acid prevents UVB-induced corneal and conjunctival degeneration through multiple effects. Invest Ophthalmol Vis Sci.

[16] Ruban VV, Anbukkarasi M, Anand T, Thomas PA, Geraldine P (2019). Oxidative stress in corneal tissue in experimental keratitis due to *Aspergillus flavus*:effect of topical voriconazole therapy. Biocatal Agric Biotechnol.

[17] Kojima T, Dogru M, Ibrahim OM, Wakamtsu TH, Ito M, Igarashi A (2015). Effects of oxidative stress on the conjunctiva in Cu,Zn-superoxide dismutase-1 knockout mice. Invest Ophthalmol Vis Sci.

[18] Sedlak L, Świerczyńska M, Borymska W, Zych M, Wyględowska-Promieńska D (2021). Impact of dorzolamide, benzalkonium-preserved dorzolamide and benzalkonium-preserved brinzolamide on selected biomarkers of oxidative stress in the tear film. BMC Ophthalmol.

[19] Farghali HA, AbdElKader NA, AbuBakr HO, Ramadã ES, Khattab MS, Salem N (2021). Corneal ulcer in dogs and cats:novel clinical application of regenerative therapy using subconjunctival injection of autologous platelet-rich plasma. Front Vet Sci.

[20] Hsueh YJ, Chen YN, Tsao YT, Cheng CM, Wu WC, Chen HC (2022). The pathomechanism, antioxidant biomarkers, and treatment of oxidative stress-related eye diseases. Int J Mol Sci.

[21] Fenollosa-Romero E, Jeanes E, Freitas I (2020). Outcome of phacoemulsification in 71 cats:a multicenter retrospective study (2006–2017). Vet Ophthalmol.

[22] Bailey K, Webb T (2022). Retrospective study of long-term outcome of phacoemulsification in 22 feline eyes with presumed congenital/juvenile cataracts (2007–2020). J Am Anim Hosp Assoc.

[23] Di Pietro S, Tabbì M, Falcone A, Macri F, Piccione G, Giudice E (2023). Hospitalization disrupts the daily rhythm of tear production in cats. Vet Ophthalmol.

[24] Sebbag L, Uhl LK, Schneider B, Hayes B, Olds J, Mochel JP (2020). Investigation of Schirmer tear test-1 for measurement of tear production in cats in various environmental settings and with different test durations. J Am Vet Med Assoc.

[25] Iwashita H, Sebbag L, Leonard BC, Saito A (2023). A review of diagnostic tests for qualitative and quantitative tear film deficiency in dogs. Vet Ophthalmol.

[26] Sebbag L, Reilly CM, Eid R, Maggs DJ (2016). Goblet cell density and distribution in cats with clinically and histologically normal conjunctiva. Vet Ophthalmol.

[27] Ewing JF, Janero DR (1995). Microplate superoxide dismutase assay employing a nonenzymatic superoxide generator. Anal Biochem.

[28] Aebi H (1984). Catalase *in vitro*. Methods Enzymol.

[29] Hissin PJ, Hilf R (1976). A fluorometric method for determination of oxidized and reduced glutathione in tissues. Anal Biochem.

[30] Percário S (2010). Prevention of oxidative stress in renal ischemia reperfusion syndrome in rats with nutritional antioxidant supplementation. Rev Nutr.

[31] Sebbag L, Kass PH, Maggs DJ (2015). Reference values, interest correlations, and test-retest repeatability of selected tear film tests in healthy cats. J Am Vet Med Assoc.

[32] Kawahara A, Utsunomiya T, Kato Y, Takayanagi Y (2016). Comparison of effect of nepafenac and diclofenac ophthalmic solutions on cornea, tear film, and ocular surface after cataract surgery:randomized trial. Clin Ophthalmol.

[33] Kim Y, Kang S, Kim S, Shim J, Go S, Seo K (2022). Reference values for selected dry eye tests in normal beagle dogs:a pilot study. J Vet Sci.

[34] Ng ATJ, Moore PA, Boveland SD (2024). Assessment of meibomian gland morphology and tear-film lipid layer using noncontact infrared meibography and meibometry, respectively, and tear-film osmolarity in healthy dogs. Vet Ophthalmol.

[35] Malmin A, Thomseth VM, Førland PT, Khan AZ, Hetland HB, Chen X (2023). Associations between serial intravitreal injections and dry eye. Ophthalmology.

[36] Eördögh R, Jakab C, Papp R, Tichy A, Nell B (2017). Density and distribution of feline conjunctival goblet cells. J Feline Med Surg.

[37] Kato K, Miyake K, Kondo N, Asano S, Takeda J, Takahashi A (2017). Conjunctival goblet cell density following cataract surgery with diclofenac versus diclofenac and rebamipide:randomized trial. Am J Ophthalmol.

[38] Macías SDCG, Jauregui Franco RO, Torres-Arellano JM, Gallegos YC, Espinosa JMM, Ríos AS (2025). Effects of ophthalmic medications on conjunctival goblet cell density in New Zealand white rabbits. Biomed Hub.

[39] Fan X, Monnier VM, Whitson J (2017). Lens glutathione homeostasis:discrepancies and gaps in knowledge standing in the way of novel therapeutic approaches. Exp Eye Res.

[40] Tomsič K, Domanjko PetričA, Nemec A, Pirman T, Rezar V, Sliskar A (2023). Evaluation of antioxidant status and lipid peroxidation in dogs with myxomatous mitral valve degeneration stage B1. Front Vet Sci.

[41] Li S, Tan HY, Wang N, Zhang ZJ, Lao L, Wong CW (2015). The role of oxidative stress and antioxidants in liver diseases. Int J Mol Sci.

